# The Lived Experience of the Trusting Nurse–Patient Relationship Among Patients Admitted to Internal Medicine Wards

**DOI:** 10.1111/jan.70344

**Published:** 2025-11-03

**Authors:** Silvia Loua Henriksen, Signe Eekholm, Ingrid Poulsen, Jimmie Kristensson

**Affiliations:** ^1^ Department of Health Sciences Lund University Lund Sweden; ^2^ Research Unit of Clinical Nursing, Department of Internal Medicine Herlev & Gentofte University Hospital Gentofte Denmark; ^3^ Department of Clinical Research Amager & Hvidovre University Hospital Hvidovre Denmark; ^4^ Department for People and Technology Roskilde University Roskilde Denmark

**Keywords:** context of care, fundamentals of care, nurse–patient relationship, nursing care, patients' perspective, phenomenology, qualitative research, quality of care

## Abstract

**Aim:**

To explore the lived experience of the trusting nurse–patient relationship among patients admitted to internal medicine wards.

**Design:**

A qualitative study guided by van Manen's hermeneutic phenomenological approach.

**Methods:**

Semi‐structured interviews were conducted with fourteen hospitalised patients in a Danish University hospital in 2024. Data were analysed through thematic reflection.

**Results:**

The analysis revealed one overarching theme—*the* *journey towards trust: bridging the need to feel safe with the pathways to wellbeing*—supported by five subthemes. Trust developed through a dynamic and interpersonal process shaped by the patient's vulnerability, the nurse's actions and their mutual understanding. When trust was present, care was experienced as coherent, meaningful and safe; when absent, it felt disconnected and inadequate.

**Conclusion:**

Trust within the nurse–patient relationship is experienced as a dynamic process shaped by the nurse's presence, authenticity and responsiveness. Rather than a static state, trust unfolds gradually, influencing how patients feel safe, understood and cared for. Relational competence should be recognised as a clinical skill, requiring the same support as technical competencies. Education, leadership and policies must protect interpersonal care. Further research should examine nurses' experiences and interventions that sustain trust.

**Implications for the Profession:**

The findings emphasise the need to recognise relational care as a professional and clinical competence. Supportive environments, relational training and organisational awareness are essential to enable nurses to prioritise trust‐based relationships.

**Impact:**

This study addresses the challenge of sustaining relational care in time‐constrained hospital settings. It offers insight into patients' experiences of trust and highlights key relational behaviours. The findings are relevant for nurses, educators and healthcare leaders aiming to strengthen relational competence and improve patient care quality.

**Reporting Method:**

The study adheres to the COREQ reporting guidelines.

**Patient or Public Contribution:**

This study did not include patient or public involvement in its design, conduct, or reporting.

## 1 Introduction

To provide high‐quality nursing care, the establishment of a trusting nurse–patient relationship is essential. Such a relationship enables nurses to recognise and respond to patients' individual needs, preferences and vulnerabilities, thereby supporting care that is clinically effective and experienced as person‐centred, dignified and emotionally safe (Allande‐Cussó et al. 2022; Kitson et al. 2013; Molina‐Mula and Gallo‐Estrada 2020; Mudd et al. 2020; McCance and McCormack 2025). Drawing on the Fundamentals of Care framework (Kitson et al. 2013), trust in nursing can be understood as a relational process in which attentiveness, empathy and respect for individuality create the foundation for therapeutic engagement. Trust has been shown to reduce patients' sense of vulnerability and to foster open communication, thereby enabling genuine collaboration between nurse and patient (Molina‐Mula and Gallo‐Estrada 2020). While the literature affirms that the trusting nurse–patient relationship is central to the provision of high‐quality care, concerns persist regarding how such a relationship is developed and sustained in clinical practice (Feo et al. 2017). There is a need for a deeper understanding of what constitutes a trusting relationship, how it is established, and what it means for the collaboration between nurse and patient, in order to inform clinical practice.

## 2 Background

The development of trust is recognised as a core element of nurse–patient relationships (Dinç and Gastmans 2013); yet, the literature does not seem to provide a clear definition of what trust in these relationships is. However, over time, concept analyses have described trust in such relationships as a relational phenomenon that involves key attributes such as empathy, respect, honesty and consistency (Allande‐Cussó et al. 2022; Johns 1996). The Fundamentals of Care framework (FoC) does however offer a guiding description of key elements that the trusting relationship should entail, such as the nurse's ability to focus on the patient, anticipate their needs and continuously evaluate their relationship (Kitson et al. 2013). Furthermore, in its updated definitions of nursing and relational practice, the International Council of Nurses emphasises trust as a foundational professional commitment and a prerequisite for effective therapeutic engagement (White et al. 2025).

Thus, the literature highlights the centrality of trusting nurse–patient relationships; however, although nurses are encouraged to establish supportive and therapeutic relationships, little evidence‐based guidance exists on how to cultivate them in clinical practice (Mudd et al. 2020) Ensuring that trusting nurse–patient relationships are an integral part of clinical practice requires knowledge that complements theoretical understandings with insights into patients' lived experiences. The patient perspective illuminates what enables trust in everyday care encounters and how nurses' actions are perceived as trustworthy. Perceived quality of care is pivotal for patients' experience of trust, and when fundamental needs are unmet, patients may feel humiliated and emotionally isolated, leading to a breakdown of trust (Bagnasco et al. 2020).

International studies into hospital care have shown that patients' fundamental needs, including hygiene, nutrition, mobilisation and communication, which are essential to high‐quality nursing care, are often not adequately met (Chaboyer et al. 2021; Mainz et al. 2025). A study exploring the prevalence of missed nursing care in Danish hospitals revealed that the nursing care tasks most frequently missed are ambulation, sufficient documentation and assisting with oral hygiene (Mainz et al. 2025). This has serious implications not only for the risk of adverse events (Ball et al. 2018) but also for the development of a trusting relationship (Bagnasco et al. 2020).

Time pressure and limited resources have been identified as key reasons why care needs within the above‐mentioned categories are not met (Jangland et al. 2018; Voldbjerg et al. 2025). Under these constraints, technical and quantifiable activities are often prioritised, while relational aspects of care, such as being emotionally present or engaging in meaningful conversations, are more likely to be deprioritised or overlooked entirely (Chaboyer et al. 2021; Mainz et al. 2025). Eekholm et al. (2021) describe how fundamental nursing care is often provided in the gaps between other more officially recognised tasks, suggesting that the relational elements of nursing may be deprioritised. As such, despite widespread acknowledgement of the importance of the relational aspect of nursing care, it appears to remain largely under‐recognised in clinical practice.

The gap between theory and practice underscores the need for inquiry into how trust emerges in everyday care. Qualitative research capturing patients' lived experiences can reveal how trust is perceived in nurse–patient interactions and generate insights to guide clinical practice towards fostering authentic connections and enhancing patient experiences.

## 3 Aim

This study aims to explore the lived experience of the trusting nurse–patient relationship among patients admitted to internal medicine wards.

## 4 Methods

### 4.1 Design

The study design is based on van Manen's hermeneutic phenomenological approach, combining both descriptive and interpretive perspectives to explore lived experience (van Manen 2013). According to van Manen (2023), the overall understanding of the lifeworld (the world as experienced by individuals in their everyday lives, separate from any philosophical or scientific analysis) is guided by existentials (existentiality, relationality, corporality, spatiality, temporality, materiality and technology), which serve as a framework for reflection throughout the analytical process. By allowing these existentials to guide the reflective process, deeper insights into the lived experience of the phenomenon, as expressed by the participants, are obtained.

### 4.2 Study Setting and Recruitment

This study was conducted in an internal medical department in a university hospital in the Capital Region of Denmark. The department receives patients referred from the emergency department who require acute treatment. It is organised into seven wards, covering four medical specialties: respiratory medicine, infectious diseases, geriatrics and endocrinology. The department has a total of 120 beds distributed across wards, with individual ward sizes ranging from 14 to 26 beds. The patient population primarily consists of elderly patients characterised by complex illness trajectories, multiple comorbidities, reduced physical and/or cognitive function and limited self‐care capacity. As such, patients often require nursing interventions that address physical, psychological and psychosocial needs. The average length of stay is 3.6 days. The department employs 188 full‐time nursing staff, of whom 133 are registered nurses (RNs) and 55 are licenced practical nurses (LPNs). In the Danish context, RNs (bachelor level nurses) and LPNs (vocationally trained nurses) both engage in direct care, though RNs hold responsibility for care planning and clinical judgement.

A purposive sampling strategy was applied. This approach enabled the identification of participants with diverse experiences relevant to the phenomenon of trust ensuring inclusion of participants able to provide rich and nuanced descriptions of the phenomenon. To be eligible for inclusion, patients had to have been hospitalised for a duration that allowed multiple interactions with different nursing staff across shifts, enabling meaningful relational development. For this reason, a minimum admission period of 24 h was set. Patients were excluded if they had communication difficulties preventing their provision of informed consent. Patients with life‐threatening or acute critical illnesses were also excluded due to ethical considerations. Eligible patients were initially informed about the study's purpose, interview format and confidentiality measures by the ward's clinical nurse specialist. After patients had given verbal permission to be contacted, the first author approached them and reintroduced the study to ensure that written informed consent was based on a clear understanding and a voluntary decision.

In accordance with van Manen's (2013) hermeneutic phenomenology, the number of interviews was not predetermined but was guided by the emergence of rich, varied accounts. Data collection ceased when further interviews no longer deepened the understanding of the phenomenon's essence. A total of 18 patients were invited to participate in this study. Four declined due to fatigue and a need to prioritise rest, while 14 patients consented to participate (Table 1). To ensure variation, two participants were included from each of the seven wards. To describe characteristics relevant to the patients' experience of the trusting nurse–patient relationship, information was collected on patients' previous experiences of hospitalisation, as well as their sex, age and functional status, as indicated by their need for home care.

**TABLE 1:** Participant information.

	*n* (%)	Mean	Range
Sex			
Male	6 (43%)	—	—
Female	8 (57%)	—	—
Age (years)		74.9	56–91
Length of hospitalisation (days)		3.9	2–6
First admission to department			
Yes	9 (64%)	—	—
No	5 (36%)	—	—
Previous hospitalisation (outside current department)			
Yes	11 (79%)	—	—
No	3 (21%)	—	—
Receives home‐based care			
Yes	6 (43%)	—	—
No	8 (57%)	—	—

### 4.3 Data Collection

Data were collected from January to March 2024 through individual semi‐structured interviews (Brinkmann and Kvale 2015) with patients during their hospital stay. An interview guide was developed to structure the interviews, containing open‐ended questions to elaborate on the phenomenon under study and to explore its nuances. Since ‘trusting nurse–patient relationship’ is a professional and theoretical term, this wording was not directly included in the interview guide. Instead, open‐ended questions asking the participant to elaborate on how trust can be evoked, what trust feels like and what it adds to the experience of being hospitalised were included. Given that patients can establish trusting relationships with any category of nursing staff and that patients often find it difficult to distinguish between RNs and LPNs, these professional roles and the differences between them from the patient's perspective were not raised in the interviews.

The data collection began with three pilot interviews conducted by the first and second authors to refine the interview guide and strengthen the interview technique. All subsequent interviews were conducted by the first author, a female PhD student. Pilot interviews were included in the analysis.

To support the participants' sense of comfort and encourage open dialogue, the interviews were conducted in a quiet and undisturbed area within the ward. If the patient had visitors and wished for them to be present during the interview, this was accommodated. On two occasions, visitors were present during the interviews, but their only contribution was to support the participants, and any comments that they made were not included in the analysis.

All participants were informed that they could pause or end the interview at any time if they felt uncomfortable or simply needed to rest. The interviews lasted between 28 and 108 min, with an average of 46 min and a total recorded interview time of 10.6 h. All interviews were audio‐recorded using a digital voice recorder and transcribed verbatim without the use of an advanced electronic system resulting in 321 pages of transcriptions. Audio recordings were downloaded to a locked drive and removed from the digital recorder. Transcriptions are stored securely in a locked facility behind a restricted‐access door.

### 4.4 Data Analysis

The analytical process was guided by van Manen's (2023) descriptions of thematic reflection and included three overall approaches to the interview transcripts: holistic reading, selective reading and detailed reading (Table 2). These readings were conducted iteratively, allowing meaning to emerge gradually from the participants' narratives. The analysis concluded with a thematic presentation of the findings. The analytical process is outlined in Table 2.

**TABLE 2:** The analytical process.

Analytical approach	Description
Holistic reading	All authors read the full set of interview transcripts multiple times to gain an overall understanding of the text and to express its eidetic meaning. The guiding question for this reading was: *What are the patient's experiences of trusting relationships with nurses?* A brief description capturing the overall meaning of the text was then formulated and discussed among all authors until a consensus was reached regarding a nuanced and comprehensive understanding of it
Selective reading	To further explore the nuances of the phenomenon under study, the following questions were asked in consideration of the text: What characterises a trusting relationship with a nurse from the patient's perspective? What is the patient's experience of how trust is established in the nurse–patient relationship? What does a trusting relationship with a nurse mean to the patient? Each interview transcript was re‐read by all authors, and meaning units that help to answer these questions were identified. Thematic descriptions of the meaning units were developed by the first author and subsequently discussed by all authors, with particular attention paid to interpretations and consistency across the full dataset
Detailed approach	The first author analysed, interpreted and grouped the meaning units into themes to deepen the understanding of the essence of the studied phenomenon. Rather than coding in a conventional sense, reflective engagement through language and thematic writing was used to reveal the essential qualities of trust in nursing care. This process involved ongoing dialogue among all the authors, refining the themes to reach a shared and nuanced understanding
Development of thematic description	A thematic description of the essence of the lived experience of the phenomenon was drafted by the first author. This was then read and discussed by all the authors to illuminate additional perspectives and deepen the interpretation and elaboration of the phenomenon. The writing and rewriting process continued until a final description that was agreed upon by all authors was generated

Applying van Manen's phenomenology requires prior experience to ensure methodological soundness. The first author has completed formal university‐level coursework in qualitative research methodologies, including phenomenology and van Manen's writings, and was supervised by co‐authors with extensive experience.

In line with van Manen's approach (2013, 2023), writing and rewriting were integral throughout the holistic, selective and detailed readings. This iterative process deepened interpretive engagement, refined thematic resonance, and ensured that the final descriptions remained closely anchored in participants' lived experiences.

### 4.5 Ethical Considerations

The study participants were interviewed while hospitalised, a situation during which they may have been physically or emotionally vulnerable. To support truly informed and voluntary consent, patients were clearly informed that participation was voluntary, responses confidential, and that the interviewer was not affiliated with their direct care or any decisions in that regard. Recognising possible power dynamics, the researcher took steps to minimise perceived authority, including not wearing a hospital uniform during interviews.

This study aligns with ethical principles outlined in the Helsinki Declaration (World Medical Association 2013). This includes safeguarding participant privacy, ensuring beneficence and upholding justice in the research process. The study was registered at the Danish Scientific Ethics Committee (journal no. F‐23078331) and the Danish Data Protection Agency through a Regional Committee (registration no. p‐2023‐15069).

### 4.6 Rigour and Reflexivity

According to van Manen (2023), the validity of a phenomenological study relies on *the soundness of the interpretive process demonstrated in the study*. The first author has extensive clinical experience in internal medicine, which informed contextual understanding and required active reflexivity to manage potential preunderstandings. Prior to data analysis, these preunderstandings were documented and subsequently revisited throughout the analytic and writing process. This supported interpretive rigour by making implicit assumptions explicit and by enabling bracketing during thematic reflections. Reflexive practices included collaborative discussions and iterative re‐reading of transcripts in light of emergent themes. All authors contributed to the interpretation through joint analytic sessions, during which findings and assumptions were critically examined to ensure transparency, coherence and trustworthiness.

## 5 Findings

The material derived from the interviews revealed various perspectives and insights into how patients perceive the trusting relationship with nurses, how trust is experienced and what trust adds to the experience of care. These perspectives and insights were described through one overall theme and five sub‐themes.

The trusting relationship begins with a simple yet profound encounter: A patient in need of help and a nurse whose role is to provide it. When help is offered, it may foster a sense of safety, but it is what unfolds within the interpersonal space—the intrapersonal exchange between the nurse and the patient—that forms the foundation from which trust can begin to emerge. However, the development of trust between the nurse and the patient does not occur automatically; it is neither a linear nor a uniform process. Therefore, the development of trust cannot be presumed, nor can it be forced. Trust unfolds gradually, as a natural gravitation towards a sense of safety and well‐being, shaped by the patient's vulnerability, need to feel safe and search for relational trust.

This individual, relational and dynamic process is captured in the main theme of the analysis: *The journey towards trust—bridging the need to feel safe with the pathways to wellbeing*. This main theme was explored through five subthemes: (1) Being vulnerable—the patient's need to feel safe in the acute phase, (2) From the essential need for safety to the search for relational trust, (3) When nursing care transcends treatment—the path from safety to trust, (4) The extra dimension of care—the experience of trust, and (5) Authenticity and sincerity—the nurses who inspire trust.

### 5.1 The Journey Towards Trust—Bridging the Need to Feel Safe With the Pathways to Well‐Being

#### 5.1.1 Being Vulnerable—The Patient's Need to Feel Safe in the Acute Phase

You can be the strongest of the strong when you're outside the hospital. And then it turns out that you are the weakest of the weak when you step through to the other side of the door [and are being admitted]. (Participant 13)

The experience of losing or lacking control is described as evoking a sense of helplessness. Control is surrendered and the individual assumes the role of a patient, entering a new and unfamiliar world that must be navigated without access to the usual skills, competencies and strategies normally relied upon to maintain a sense of stability. The ability to support oneself appears diminished, which could lead to feelings of fear. One patient explained: *‘Well, I am constantly nervous and trying to stay in control. So, I keep writing everything down. (…) It's simply because I feel unwell’ (Participant 7)*. This attempt to maintain control highlights how the loss of stability during hospitalisation can heighten anxiety. Not being able to rely on one's own resources or achieve a sense of safety left patients feeling insecure and vulnerable. In such moments, achieving a feeling of safety was outside the patient's control and depended on the help of others. This dependence made the need to feel safe particularly crucial in the stage soon after admission—the acute phase of hospitalisation.

The patients expressed a sensation of needing to feel safe within their body, a need to understand what is happening and a need to experience being cared for. The space in time in which help is still awaited is associated with feelings of vulnerability, insecurity and fear. When the nurse demonstrates control over the situation, relieves the patient's suffering and shows compassion, patients describe that a feeling of safety is evoked.

On top of everything I've been through, it's like… well, now I can finally relax a little. Now I'm in good hands, because I wasn't before. (…) Now there's someone taking care of me. Now I don't have to figure everything out on my own. (…) Now I'm allowed to just be ill. (Participant 3)

Beyond individual encounters, patients also drew on a more fundamental trust in the healthcare system. Before any personal interaction with staff, they believed that help and safety would be available within the hospital setting. Nurses therefore became the first concrete embodiment of this institutional trust. These encounters could either reinforce patients' expectations or profoundly undermine them, with participants describing how their initial trust was intensified or, in some cases, dramatically weakened during these early interactions.

Patient: She didn't listen to me. I said: ‘You have to take it out [the peripheral IV catheter], it hurts’. She didn't listen to me; she just said, ‘Well, you need fluids’.

Interviewer: How did that make you feel?

Patient: Powerless… I just had to hope the next shift would help me. (Participant 12)

#### 5.1.2 From the Essential Need for Safety to the Search for Relational Trust

She kind of motivates me to feel a bit happier, in some way. (…) When someone says: ‘How about I bring you a nice hot cup of coffee? Then you can enjoy that for a bit’. Then you're like, oh, lovely. It's like being wrapped in a blanket. (Participant 3)

As the acute phase stabilises and patients become accustomed to their hospitalisation, their needs begin to shift. The intense focus on safety is no longer described with the same urgency. Instead, what becomes important is closely connected to patients' sense of well‐being and to the nurse's ability to evoke or support it.

One patient contrasted the experience of professionalism in the emergency department with that of the medical ward: *‘They need to have a special team in the emergency room, they were so professional, it was amazing. Up here [in the medical ward], it's more care and medication, isn't it?’ (Participant 4)*. In this perspective, nurses' professionalism was tied to safety in the acute phase, while in the medical ward the emphasis shifted towards interpersonal care.

The interpersonal relationship between the nurse and patient is explored, not as a need but rather as a possibility. This is revealed through descriptions illustrating that the patients do not view interpersonal trust in the nurse–patient relationship as something on which they depend to feel safe. However, feeling safe with the nurse allows the seeds of trust between them to be sown and potentially grow.

When I was admitted, no one told me what was wrong with me. No one told me whether it was something to worry about or if it was normal. And if I'm to feel safe—not trust, but safe—well, then someone should have said: ‘A lot of people have this, it's nothing to worry about’. Then I would've just relaxed and not thought more about it. But now I'm lying here thinking whether I can find some information about it myself, instead of someone just telling me. (Participant 7)

Nurses' actions that are perceived as expressing a genuine desire to help contribute to a sense of well‐being. This becomes especially apparent when such actions clearly reflect that supporting the patient is the nurse's core intent and purpose in providing care. Situations described in this context are characterised by the patient feeling that the nurse is going ‘the extra mile’: doing something unexpected, something not directly needed, yet something that indeed feels good and something solely done for the sake of the patient's sense of well‐being. These small acts made patients feel seen, supported and cared for.

#### 5.1.3 When Nursing Care Transcends Treatment—The Path From Safety to Trust

When they told me that they had been sitting with me during those days in there [when I was in and out of consciousness], I thought, ‘Wow, these are complete strangers’. And imagine*—*they actually wanted to do that. That's when the trust came. I thought, wow, they really did take care of me. (Participant 2)

The encounter between the patient and the nurse is described by some patients as if they become closer through experiencing shared space. It is within this lived space that trust between them can develop. The patients experience nurses' actions and sense the emotions they evoke. Actions highlighted by the patients included nurses offering a warm welcome and introducing themselves by name, as well as initiating and maintaining conversation by asking relevant and attentive questions. As one patient explained: *‘There are those who just do what they need to and leave and those who stay a bit longer and talk. They don't leave as fast’ (participant 1)*. The way the nurse indicates an interest in talking with the patient, beyond the time spent on the task in focus opens a possibility of a shared space between them.

This possibility of shared space was also evident when a nurse combined a practical task with personal engagement. One participant recalled how a nurse, while assisting her with eating, asked to sit beside her and initiated a conversation, shaping a moment where the nursing task transcended its immediate purpose and created a sense of comfort.

Providing care through these small, benevolent actions makes the patient feel comfortable, through which the patient experiences trust being developed. Through these actions, the nurse is also perceived as being intent on helping and supporting the patient's recovery and promoting a sense of well‐being during their stay in the hospital. The patient's need to feel safe and experience well‐being is felt as a shared aim between the nurse and the patient, and a sense of unity and cooperation in pursuing this aim creates trust in the relationship between them.

Some patients experienced the dialogue between nurse and patient as a way out of an unfamiliar and helpless state and a path towards trust. However, this presupposes that the patients experience such conversations as reflecting the nurse's ability to express understanding and a sincere desire to help through actively listening and engaging in the conversation.

I get that the nurses are super‐busy. But it's a bit like being at a restaurant where they ask, ‘Does the food taste good?’ and before I even get to answer, they've already walked away. Then don't ask. And if I actually say that it doesn't taste good, they reply, ‘That's great’. I mean, they didn't even hear what I said. So don't ask me, if you don't care about the answer. (Participant 14)

When two individuals meet, small talk can bring them together in the conversation. Similarly, conversations and small talk between nurses and patients can bridge the gap between them and provide a way for them to approach each other. The conversations bring them together. When they steer away from talking about the medical context or related subjects and their conversation shifts to more fundamental aspects of life, they meet in a neutral space, as equals. This conversation is the basis for the meeting of two individuals and the foundation for trust. Engagement in such conversations shows the nurse's interest in the patient as an individual, not only as a patient. When the nurse asks about the patient, is curious and takes the initiative to start a conversation and be open to dialogue, it is experienced as a sincere and genuine interest in the individual.

The way she responded to me the first time… It was completely natural. And then the conversation just started. And I could feel that, when I said something, she understood my concern and brought it up again herself. It was she who brought it up again. Not me. It was she who returned to it. (Participant 11)

Thus, the informal nature of such small talk is perceived as creating a space and atmosphere for patients to be both open and share a bit of themselves. When patients sense that the nurse is also open and demonstrates interest and willingness to engage, the interest in the relationship is experienced as being reciprocated. The nurse's ongoing and repeated engagement in conversations establish fundamental trust in the relationship.

The mutual understanding developed between nurse and patient also establishes a balance in their relationship. Both have expressed vulnerability, and they meet in this shared experience. Trust builds a bridge between them. Trust disarms the patient and creates a place that is safe to be vulnerable. Once this is established, the patient can let go and focus on ‘just’ being a patient attending to personal needs rather than trying to navigate and manage the unfamiliar world of the hospital.

I miss her because she… she made it easy to be a patient when she was here. When someone listens. Or someone takes care of you. [When someone sees] that I'm a human being. That I'm not just a piece of meat lying here in the bed. That's what made the difference. That… I felt that… that I… that I mattered… I don't really know how to explain it better (…) Just like I had respect for her, she also had respect for me. (Participant 10)

Some of the patients expressed that if they sense a void in the shared space with the nurse, a way of filling it is by taking responsibility for initiating a conversation or attempting to engage with the nurse. In these situations, the nurse holds the power to decide whether to accept the patient's invitation and establish a relationship.

Then there's the other group. I don't feel good about them. I just note that they're doing a job. (…) Talking to them doesn't lead anywhere. They don't get anything out of it, either. And they're not interested, by the way—that's very important. They just do the task and then they leave again because they have to move on to the next one. (Participant 1)

An equal, respectful and curious dialogue is perceived by the patients as an expression of the nurse's genuine interest in understanding how to provide the best possible care, fostering trust and a feeling of being safe in the situation. Conversely, if communication is experienced as rudimentary or false or if the nurse fails to involve the patient in the dialogue, the patient is left feeling abandoned and forced to interpret and navigate a complex and unfamiliar situation alone, potentially leading to feelings of anger and powerlessness.

#### 5.1.4 The Extra Dimension of Care—The Experience of Trust

She's the only one who noticed it. And said: ‘You need clean clothes. We'll take care of that’. I even got a long‐sleeved shirt too, on top of it. (…) She sees that. She notices things. There are some people who actually see things. (Participant 1)

Some patients found it difficult to describe precisely how trust within the nurse–patient relationship was experienced, expressed or manifested. Trust was often portrayed as existing in the tension between a concrete action and the abstract feeling it evoked, the two aspects being inseparably intertwined. It could not be detached from the patient's overall experience but became apparent through the holistic experience of what the nurse did and how it was done. When patients sought to put into words how trust emerged, they linked nurses' actions to values such as empathy, compassion and sincerity. The patient's perception of these values arose from behaviours that appeared to embody them, shaping the patient's experience of trust—behaviours that made the values implicitly felt rather than explicitly stated. In this sense, trust was inseparable from the nurse's actions: what was said, when it was said, and how it was expressed. It was the accumulation of such small encounters that together formed the experience of trust.

It's like she just goes that extra mile (…) She says, ‘Should I get you a nice hot cup of coffee? Or what would you like? Would you like a hot cup of tea? You could have both. And how about some grapes?’ That's how she is—all the way through. She tries to find little treats (…) She really engages and kind of tries to lift you up, in some way. (Participant 3)

The ways in which trust affected collaboration and interaction between nurses and patients were not easily defined or described by the patients. For the patient, it felt natural—the interaction between the two, within the relationship, is natural. Cooperation and communication occur with ease and feels unforced. Trust creates a sense of being understood, boosting the patient's motivation to perform self‐care and health‐promoting activities such as eating, getting out of bed and engaging in acts of personal wellbeing, as well as the concrete desire to return home. The trusting relationship between nurses and patients enhances the experience of recovery and can even evoke a sense of physical relief through the experience of care. For some, trust even made the difference between staying in hospital or leaving prematurely: *‘If I hadn't trusted them [the nurses], then I'm a bit afraid that I would've gone home. Then I wouldn't be sitting here now’ (Participant 2).*

The experience of trust within the relationship added a special dimension to the nursing care provided. Acts of care provided by a nurse with whom a trusting relationship has not been established are not perceived as complete compared with when the same acts are carried out within the context of a trusting relationship.

There are also some nurses who are so sweet, buzzing around, smiling [and asking] (…) ‘Would you like fresh bed linen, would you like your…?’ But they don't get what really matters. (Participant 11)

The relationship with the nurse shapes how patients experience and value the care they receive. Trust makes nursing care distinctive, as it arises from a fusion of action and behaviour—what is done, how it is done, and the values perceived by the patient.

#### 5.1.5 Authenticity and Sincerity—The Nurse Who Inspires Trust

You know that thing they call the X‐factor, right? Well, that's what she has. (…) There's just something about her. I mean, she just has that… She's always trying to be a little nurturing, a little extra caring. That's just how she talks all the time. ‘Hi, now let's take a look, and…’ The other nurse doesn't do that. She doesn't talk like that. (Participant 3)

Patients described that each nurse–patient relationship was experienced differently, but these differences were not necessarily seen as positive or negative. Instead, they provided a basis for comparison, highlighting what was unique and distinctive in relationships founded on trust. Nurses who were perceived as trust‐inspiring were described as possessing a particular quality that was intangible and difficult to put into words—a way of being that appeared so natural it could not be learned or adopted. It is described as a genuine and sincere ability to make the patient feel seen as an individual—a social and empathic competence that makes possible an encounter between two individuals founded on understanding and respect. This quality is experienced as something so distinctive that it appears to be a factor almost completely determined by one's personality or is even innate: you either have it or you do not.

When you are a nurse, some part of the genes has to be the ‘care’ gene. They have to like taking care of people and listening to people (…) That's what they need to have within. That they actually enjoy caring and making sure that the person is doing well and gets better. (Participant 11)

This way of being was often described in terms of authenticity—the sense that the nurse was ‘real’. Patients emphasised that such authenticity could not be performed or imitated but reflected who the nurse fundamentally was. When harmony was perceived between what the nurse did and how it was done, trust was strengthened.

Authenticity was closely tied to sincerity, which patients recognised when nurses demonstrated a genuine desire to help and understand. This sincerity was often conveyed through curiosity and openness, reflected in actions such as asking questions, expressing wonder and meeting the patient on their own terms.

I don't like having my blood drawn, and the nurse who took my blood sample said that she actually didn't like it, either. And that was actually very honest, instead of saying something like, ‘just pull yourself together’. But it's just hard for me and I thought it was nice that she gave a bit of herself. Something genuine. (Participant 14)

For patients, the perception that the nurse was primarily motivated by a genuine interest in helping was essential to the experience of trust and, consequently, to the quality of the relationship. Some nurses could offer patients a considerable amount of attention and ask about their needs but still fail to grasp what truly mattered. Others initiate conversation but give the impression of not really listening. When the patient perceived harmony between what is done and how it is done, the nurse's authenticity becomes evident, reinforcing the distinctive experience of trust in the nurse–patient relationship.

## 6 Discussion

### 6.1 Trust in Nursing Care

The findings reveal that trust within the nurse–patient relationship develops through a sensory and dynamic process that takes place within the interpersonal encounter shaped by the patient's overall impression of the nurse's intentions, behaviours and presence. When patients perceive that a nurse's actions are grounded in a sincere and genuine wish to help, these actions are experienced as credible and authentic. It is exactly this that is experienced as the foundation of a trusting relationship and the path towards the delivery of high‐quality nursing care.

Within the Fundamentals of Care (FoC) framework, Kitson et al. (2013) identified the trusting nurse–patient relationship as the starting point for integrating the physical and psychosocial dimensions of care. More recent work reinforces this position: McCance and McCormack (2025) describe relational engagement, mutual respect and authentic presence as core components of person‐centred care, each of which fosters the conditions for trust to emerge. Similarly, Pene et al. (2025) emphasise that trust is not a peripheral attribute but a prerequisite for delivering fundamentals of care in ways that enable patients to feel safe, valued and understood. This is confirmed in this study: When patients feel recognised as individuals and when nurses demonstrate an understanding of their needs and situation, care is experienced as holistic and characterised by compassion. Conversely, when trust is lacking, patients perceive nurses as less able to identify and respond to their needs. Thus, it is not merely the actions themselves but the way they are delivered and linked to the patient's lifeworld that shapes the quality of the care experience. Previous research has highlighted that in the absence of relational anchoring, nursing care risks becoming fragmented and reduced to simply a series of tasks (Kitson et al. 2014). The present study supports the view that it is through a trusting relationship that high‐quality nursing can be achieved.

The development of a trusting nurse–patient relationship is inherently complex and unpredictable, yet it plays a central role in how care is perceived and received. This underscores a significant point: the trusting relationship should not be understood as a static entity or outcome but as a dynamic phenomenon. By recounting and analysing patients' lived experiences, this study illustrates how trust contributes to feelings of safety and coherence and highlights its essential role in patients' experiences of care. These insights are critical for the development of a nursing practice in which this relationship is recognised as an active and integrated component of care that requires professional competence and the capacity to reflect on patients' situations and needs and adapt accordingly.

### 6.2 The Trust‐Inspiring Nurse

Trust in the context of nursing care is supported by the nurse's ability to demonstrate empathy, presence and attentiveness (Feo and Kitson 2016). Small but significant actions—such as making eye contact and introducing oneself—are described as fundamental to establishing a trust‐based relationship (Mikkelsen et al. 2019). Trust is regarded as an integral aspect of human interaction, and only actions fostering mistrust can threaten it (Martinsen 2010).

However, the findings of this study indicate that the development of trust is more complex. This highlights a gap between theory and practice, where the theoretical approaches often outline ideals for how the nurse–patient relationship should be; the reality is experienced as far more dynamic, nuanced and emotionally charged.

Patients engage in an unconscious and sensory assessment of whether the nurse appears engaged, authentic and reachable. It is a holistic impression—rather than isolated words or actions—that determines whether trust can emerge and the relationship can evolve. Johns' (1996) concept analysis confirms that trust is built through the patient's continuous evaluation of the nurse's competence and intentions, often based on body language and emotional cues. Similarly, McCabe (2004) demonstrates that nonverbal communication—tone of voice, eye contact and body language—strongly influences patients' perceptions of sincerity and empathy. Trust is thus developed not only through the spoken word but through tone, presence and subtle cues. In other words, the trusting relationship is shaped quietly—through atmosphere and presence.

A central theme in patients' descriptions of trust in their relationships with nurses is authenticity. Nurses perceived as genuine and as ‘being themselves’ are perceived as trust‐inspiring. Previous research confirms the significance of authenticity in the development of trust. Pratt et al. (2021) argued that authenticity not only encompasses being true to oneself but also involves active engagement, sincere interest, appropriate self‐disclosure and being present when communicating. Participants' descriptions of nurses who offered comfort, listened attentively and expressed warmth resonate closely with the concept of compassion in nursing. These actions, while often subtle, appeared to foster a sense of emotional safety and contributed significantly to the development of trust. As such, the experience of compassionate care may be considered a fundamental dimension in the formation of trust‐based relationships (Bond et al. 2018).

However, van Belle et al. (2020) noted that not all nurses—often despite good intentions—succeed in demonstrating the presence required to foster trust. Similarly, the present findings show that the qualities of nurses who inspire trust are often perceived as a personal quality, something innate rather than acquired. This perspective is reflected in the findings of Wiechula et al. (2016), which show that patients often perceive nurses who appear trustworthy as individuals possessing innate or personal characteristics, rather than qualities developed through professional training.

This raises the question of whether relational competence is a personal trait or a professional skill. Viewing it as an innate characteristic undermines the potential for developing or refining it. However, research indicates that emotional intelligence and an awareness of the centrality of relational aspects within the holistic context of care are crucial to patients' experiences and can be cultivated and supported (Jangland et al. 2018; Khademi et al. 2021).

Establishing and maintaining a trusting nurse–patient relationship is a challenging task; being able to offer the attention, understanding and foresight needed to build trust requires both intellectual and emotional skills (Kitson 2018). This underlines that the capacity to foster trust within the nurse–patient relationship, must not be seen as reflecting one's personality, but rather as a professional capability.

### 6.3 The Nurse–Patient Relationship in Context

Findings from this study indicate that time pressure in clinical contexts can create distance in nurse–patient relationships. Patients link busyness with reduced presence; when nurses appear absent or hurried, patients are less able to develop trust in them. In time‐constrained settings nurses must inevitably prioritise particular tasks required of them. Research on the parts of nursing care that are often overlooked has shown that relational aspects—such as communication, emotional support and presence—are frequently deprioritized (Chaboyer et al. 2021; Mainz et al. 2025).

Feo and Kitson (2016) argued that nurses' clinical priorities may be shaped by organisational and cultural forces that emphasise the value of efficiency and biomedical tasks. Within such systems, relational aspects of care may be perceived as less valuable, which can lead to their routine marginalisation in practice. Feo and Kitson (2016) further suggested that such cultures influence not only what is prioritised but also how nurses come to understand and value their role. This indicates that there is a risk of fundamental care—including presence, communication and emotional support—being consistently overlooked, despite its recognised importance in nursing theory and for optimising the patient experience.

Bridges et al. (2013) argue that relational care is not necessarily deprioritised due to a lack of willingness on the part of nurses, but rather as a coping strategy in response to the moral distress that arises when time and resource constraints prevent full engagement in the nurse–patient relationship. In such contexts, the emotional and interpersonal aspects of care are often the first to be compromised, potentially weakening the relational foundation upon which trust is built. However, if relational care is the first to be sacrificed in practice, despite being theoretically positioned as fundamental to quality nursing care, a professional and ethical dilemma emerges.

While time pressure is a real and persistent barrier, the findings of this study suggest that patients interpret trust primarily through the nurse's attitude, presence and attentiveness, rather than through the duration of time spent. Small gestures—such as a calming, reassuring tone or a moment of undivided attention—were experienced as profoundly meaningful. This perspective is supported by the Person‐Centred Nursing Framework developed by McCormack and McCance (2006), in which sympathetic presence, engagement and respect for patients' values and beliefs are identified as central relational processes that contribute to person‐centred care. These elements demonstrate that trust‐building care can be enacted even within the constraints of busy clinical settings, when nurses are supported to bring relational intention into their everyday practice.

Mudd et al. (2023) found that nursing leaders often lack clear strategies to support relational care. Despite a broad consensus on the importance of the nurse–patient relationship, this recognition is rarely translated into specific organisational tools or strategies. Relational care is frequently described in abstract terms, without the development of corresponding methods for implementation, practice or evaluation. Without appropriate managerial support, it becomes difficult for nurses to prioritise or refine the relational aspects of their practice.

Evidence suggests that leadership plays a critical role in creating a culture that supports the nurses' relational competencies. Person‐centred leadership highlights the ward manager's role in role modelling relational practices and, through everyday leadership, shaping a culture of care (Cardiff et al. 2020). Leadership behaviours that promote staff wellbeing and embed person‐centred values into routine care can create the conditions for relational competence to thrive. This is further supported by Lynch et al. (2018), who highlights that person‐centred leadership is not only about what leaders do, but also how they are and how they embody relational values in their interactions and decisions. This leadership presence enables staff to engage more fully in relational care, integrating it into the everyday texture of clinical practice rather than treating it as an optional ideal.

### 6.4 Strengths and Limitations

A key strength of this study is its phenomenological design, which enabled an in‐depth exploration of patients' lived experiences of trust in the nurse–patient relationship (van Manen 2013, 2023). The use of van Manen's hermeneutic phenomenological approach provided a strong methodological foundation for uncovering the existential and relational aspects of trust within hospital care. Moreover, conducting interviews with patients when they were hospitalised provided access to their experiences in real time, offering rich, contextually situated insights into how trust is formed and perceived in everyday care encounters.

This study benefited from variations in the characteristics of the participants. This heterogeneity contributed to the richness of the data and enabled diverse expressions of trust to emerge. Additionally, the analytical process was conducted iteratively and collaboratively by all authors, which supported deep and reflexive engagement with the material.

While these features strengthened the study, certain limitations should be noted. The focus on a single medical department within a Danish university hospital allowed for a coherent and contextually rich account, but the findings are situated within this specific setting and healthcare system. Additionally, patients were not asked to differentiate between RNs and LPNs, which means that potential differences in how trust is established across professional roles were not explored.

Another important limitation concerns the vulnerability of patients during hospitalisation. Physical or emotional strain may have impacted their ability or willingness to articulate certain experiences or refuse to participate in the study. While several ethical precautions were taken to counter this, research involving vulnerable populations remains susceptible to potential power imbalances between the subjects and the researchers.

The first author's prior experience as a nurse presented both a strength and a potential limitation in the study setting. While this background facilitated rapport and contextual insight, it posed a potential risk of interpretive subjectivity. Although preunderstandings were articulated and reflected upon throughout the research process and this reflection was supported through ongoing dialogue within the research team, the influence of the researcher's perspective on the interpretive process cannot be entirely eradicated.

### 6.5 Recommendations for Further Research

Further studies are needed to explore how nurses themselves experience the relational dimension of care in clinical practice. Research should also examine how relational competence can be cultivated and sustained. Finally, there is a need to develop and evaluate interventions that explicitly support the formation of trust‐based relationships in clinical settings.

### 6.6 Implications for Policy and Practice

This study highlights that trust is not an optional or supplementary element in nursing care but rather a fundamental condition for providing meaningful, individualised care. By deepening the understanding of how patients experience and interpret trust in the nurse–patient relationship, the obtained findings can guide clinical practice in fostering meaningful connections, improving patient experiences and enhancing care outcomes. However, this requires the trusting nurse–patient relationship to be seen not as a theoretical ideal but as a professional standard enabled by educational programmes that include relational competence as a core learning objective, promoting reflection and authenticity in nurse–patient interactions. It also requires that nursing leaders actively support the relational dimensions of nursing by acknowledging them as essential to quality healthcare and creating conditions where presence, attentiveness and interpersonal engagement are prioritised, even in busy clinical settings. It is essential to recognise the influence of political and systemic pressures that shape clinical cultures and priorities and actively resist these forces when they compromise relational care. Nursing leaders have a key role in advocating for professional standards that protect the time and space needed for meaningful nurse–patient interactions.

## 7 Conclusion

This study illuminates trust as a dynamic process shaped by the nurse's presence, authenticity and capacity to respond meaningfully to the patient's situation. Rather than a static state, trust unfolds gradually, influencing how patients feel safe, understood and cared for.

The findings underscore that relational competence should be recognised as a clinical skill, requiring the same organisational support as technical and procedural competencies. Educational programmes must foster reflective practice and relational awareness, while healthcare leaders should ensure that organisational priorities protect time and space for interpersonal care. Policies that acknowledge relational engagement as fundamental to quality and safety are essential to sustaining trust‐based nursing practice.

Further research should explore nurses' experiences of relational work and develop interventions that explicitly support trust‐building in clinical settings. Such inquiry can inform professional strategies to sustain the trusting nurse–patient relationship as a prerequisite for high‐quality nursing care.

## Author Contributions

S.L.H., S.E., I.P. and J.K. made substantial contributions to conception and design, or acquisition of data, or analysis and interpretation of data. S.L.H., S.E., I.P. and J.K. involved in drafting the manuscript or revising it critically for important intellectual content. S.L.H., S.E., I.P. and J.K. given final approval to the version to be published. Each author should have participated sufficiently in the work to take public responsibility for appropriate portions of the content. S.L.H., S.E., I.P. and J.K. agreed to be accountable for all aspects of the work in ensuring that questions related to the accuracy or integrity of any part of the work are appropriately investigated and resolved.

## Ethics Statement

The study is registered at the Danish Scientific Ethics Committee (journal no. F‐23078331) and the Danish Data Protection Agency (registration no. p‐2023‐15069).

## Consent

Written informed consent was obtained from all participating patients.

## Conflicts of Interest

The authors declare no conflicts of interest.

## Data Availability

The authors have nothing to report.
